# Is enhancing the professionalism of healthcare providers critical to tackling antimicrobial resistance in low- and middle-income countries?

**DOI:** 10.1186/s12960-020-0452-7

**Published:** 2020-02-11

**Authors:** Mishal S. Khan, Sothavireak Bory, Sonia Rego, Sovanthida Suy, Anna Durrance-Bagale, Zia Sultana, Sophea Chhorn, Socheata Phou, Chanra Prien, Sotheara Heng, Johanna Hanefeld, Rumina Hasan, Vonthanak Saphonn

**Affiliations:** 1 Faculty of Public Health & Policy, London School of Hygiene & Tropical Medicine, 15-17 Tavistock Place, London, WC1H 9SH United Kingdom; 2 Aga Khan University, Karachi, Pakistan; 3 Faculty of Pharmacy, University of Health Sciences, Phnom Penh, Cambodia; 4 Department of Public Health, University of Health Sciences, Phnom Penh, Cambodia; 5 University of Health Sciences, Phnom Penh, Cambodia; 6 Faculty of Infectious and Tropical Diseases, London School of Hygiene & Tropical Medicine, London, United Kingdom

**Keywords:** Medical education, Professionalism, Cambodia, Pakistan, Antimicrobial resistance

## Abstract

**Background:**

Healthcare providers’ (HCPs) professionalism refers to their commitment and ability to respond to the health needs of the communities they serve and to act in the best interest of patients. Despite attention to increasing the number of HCPs in low- and middle-income countries (LMIC), the quality of professional education delivered to HCPs and their resulting professionalism has been neglected. The Global Action Plan on Antimicrobial Resistance (AMR) seeks to reduce inappropriate use of antibiotics by urging patients to access antibiotics only through qualified HCPs, on the premise that qualified HCPs will act as more responsible and competent gatekeepers of access to antibiotics than unqualified HCPs.

**Methods:**

We investigate whether weaknesses in HCP professionalism result in boundaries between qualified HCPs and unqualified providers being blurred, and how these weaknesses impact inappropriate provision of antibiotics by HCPs in two LMIC with increasing AMR—Pakistan and Cambodia. We conducted 85 in-depth interviews with HCPs, policymakers, and pharmaceutical industry representatives. Our thematic analysis was based on a conceptual framework of four components of professionalism and focused on identifying recurring findings in both countries.

**Results:**

Despite many cultural and sociodemographic differences between Cambodia and Pakistan, there was a consistent finding that the behaviour of many qualified HCPs did not reflect their professional education. Our analysis identified five areas in which strengthening HCP education could enhance professionalism and reduce the inappropriate use of antibiotics: updating curricula to better cover the need for appropriate use of antibiotics; imparting stronger communication skills to manage patient demand for medications; inculcating essential professional ethics; building skills required for effective collaboration between doctors, pharmacists, and lay HCPs; and ensuring access to (unbiased) continuing medical education.

**Conclusions:**

In light of the weaknesses in HCP professionalism identified, we conclude that global guidelines urging patients to only seek care at qualified HCPs should consider whether HCP professional education is equipping them to act in the best interest of the patient and society. Our findings suggest that improvements to HCP professional education are needed urgently and that these should focus not only on the curriculum content and learning methods, but also on the social purpose of graduates.

## Background

The urgent need to increase the number of healthcare professionals (HCPs) in low- and middle-income countries (LMIC) has been highlighted by international agencies and has captured the attention of some LMIC politicians [1–5]. Thus, in recent decades, there has been an increase in the number of HCPs entering the workforce. Globally, it is estimated that 400 000 doctors and 550 000 nurses/midwives graduate every year [6]. However, the quality of professional education delivered to HCPs, and the social impact of the increased number of HCPs, has received much less attention [7]. For instance, the increased number of HCPs does not address concerns about major gaps in their knowledge and professionalism, including technical skills and essential values and behaviours that enable them to benefit society [1, 6, 8]. In LMIC, these concerns are driven by evidence on variations in class sizes, questionable examination procedures, urban bias in the location of HCP education institutes, and the lack of regulation over teaching content, methods, and standards [6, 9–11].

HCP commitment and ability to respond to the health needs of the communities they serve and to act in patients’ best interest is critical for numerous reasons. It has a major impact on the quality and cost of care that patients receive during transactions characterised by uncertainty, informational asymmetry, and buyer vulnerability [12]. Beyond impacts on individuals, the education and role of HCPs affect global health security owing to the increasing cross-national transfer of health expertise, health services, and infectious diseases [13, 14]. For example, in the United States of America, Canada, and most nations in Western Europe, a quarter of physicians acquire medical training outside the country they practise in [6]. Finally, and most closely related to aims of this study, it is essential to take into account the professionalism of HCPs when formulating global health guidelines that rely on the technical and ethical competence of HCPs in LMIC for successful implementation.

The Global Action Plan on Antimicrobial Resistance (AMR)—adopted by the membership of the World Health Organization (WHO), the Food and Agriculture Organization of the United Nations, and the World Organisation for Animal Health in 2015—is a salient example of such a global guidance document. LMIC are currently attempting, with some challenges, to implement it [15, 16]. A key pillar of the Global Action Plan on AMR is optimising the use of antimicrobial agents, and the guidelines urge patients to access antimicrobials only through qualified HCPs. The implicit and critical assumption behind this strategy is that qualified HCPs will prescribe or dispense antibiotics responsibly and that directing patients towards qualified HCPs, and away from unqualified HCPs, will reduce inappropriate use of antimicrobials [15]. However, when HCP professionalism is lacking, boundaries between qualified HCPs and unqualified providers can be blurred [17, 18]; for example, qualified providers may routinely fail to follow treatment guidelines [19] or regulations around prescribing [20], and informal providers may demonstrate more empathy towards patients than qualified HCPs [17, 21]. We therefore hypothesise that weaknesses in the professionalism of HCPs—including doctors, pharmacists, and nurses—will have an impact on inappropriate use of antibiotics, and we investigate this in two LMIC, Pakistan and Cambodia.

### Professionalism and social accountability of HCPs

**Fig. 1:**
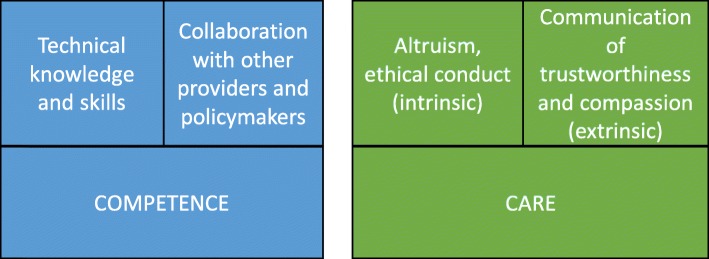
Components of HCP professionalism

There is some variation in the conceptualisation of professionalism of HCPs among scholars, with differences centred around whether professionalism is connected to HCPs’ inner morality or to observable behaviours or to integration into a community of practice [22]. Two core attributes can be identified across conceptualisations, which we term competence and care [22–26]. Competence encompasses both the technical knowledge and clinical skills to provide high-quality care, and the ability to collaborate with relevant HCPs and policymakers to ensure that the health system works optimally. Care includes two distinct components, intrinsic and extrinsic. The intrinsic component centres on altruism and ethical conduct, including a ‘commitment to placing the interests of the patient ahead of those of the professional’ [27]. The extrinsic component is a demonstration to the patient and their caregivers, through effective communication, of empathy, trustworthiness, and compassion such that the patient has confidence in the HCP’s advice. We summarise our conceptualisation of the components of HCP professionalism in Fig. 1.

There are opposing views on the goals of HCP professional education. One view is that HCP education is a public good, designed to develop a socially responsive health workforce that meets the health needs of the communities it serves. This could encompass producing graduates that not only have relevant knowledge, skills, and values but also the drive to influence the system in a positive direction. However, in some contexts, HCP education is increasingly seen as a private good geared towards helping those who make substantial investments to pay for the lengthy postgraduate qualification to maximise social mobility and personal success [28, 29]. Concerns have been raised about HCP education focusing too closely on the acquisition of biomedical information that directs students away from developing the skills and attitudes required to address the determinants of health in their patients. However, innovations and improvements have concentrated on curriculum content and learning methods, rather than on the social purpose and moral obligations of the curriculum [11].

## Methods

### Study setting

We selected Pakistan and Cambodia for this study because the countries differ in terms of socio-cultural context and size, but are experiencing similar dynamics with respect to the evolution of the health and HCP education systems. Pakistan, a predominantly Muslim country in South Asia, is the sixth most populous in the world, and Cambodia, located in the southern portion of the Indochina Peninsula in Southeast Asia, has a small, predominantly Buddhist population of approximately 15 million [30]. The majority of patients in Cambodia and Pakistan seek care from private HCPs, with approximately 60–65% paying out of pocket charges for healthcare [31–33]. In both countries, HCP education is increasingly provided by private institutions, and existing public sector institutions are typically under-resourced [34]. This partly reflects a low investment in health, including HCP education; 6% of Cambodia’s GDP and 3% of Pakistan’s GDP are invested in health [35]. Curricula used in HCP education are criticised for not being updated frequently enough, having been influenced by the colonisation of Pakistan and Cambodia by the British and French, respectively [36]. We were able to examine whether these similarities in underlying systemic issues manifest consistently in the professionalism of HCPs even when socio-cultural features differ. Here, we provide a brief overview of relevant contextual factors in our study countries.

#### Cambodia

The current state of health and health professional education has been drastically shaped by the systematic killing of intellectuals—including qualified HCPs—during the Khmer Rouge regime and the consequent civil war [37, 38]. In 1979, only 45 medical doctors, 26 pharmacists, 28 dentists, and 128 medical students had survived the Khmer Rouge regime [39]. After the genocide, no professors, of any faculty, remained in Cambodia, and medical, nursing, and pharmacy training, which was fairly comprehensive until this period, was severely disrupted and is still recovering [34, 40]. Cambodia’s leading public medical school, the University of Health Sciences, resumed operation in 1980.

There are 18 HCP educational institutions in Cambodia. There are two public medical schools, and a Ministry of Health report from 2018 indicates that there are three private medical schools, although news reports suggest that the number is higher, as several private medical schools have opened in recent years [41, 42]. Entrance exams for medical schools have recently been the subject of controversy and protests, as the process to reduce bribery and cheating has resulted in fewer students passing the exams [43, 44].

A basic medical qualification is granted after 6 years of general medical education and 2 years of clinical rotations across four disciplines (general medicine, surgery, gynaecology, and paediatrics). Upon completion, students are awarded an MD. To become qualified to practise medicine legally, doctors must pass the national exit examination and register with the Cambodian Medical Council (CMC). Registration with the CMC lasts for between 1 and 3 years depending on the category of doctor, and re-registration requires completion of some continuing medical education (CME) courses. The extent to which these registration requirements for doctors are enforced is unclear; the CMC website ‘strongly encourages’ timely registration but does not mention specific penalties for failure to do so.

A pharmacy degree takes 5 years, and students must pass a national licencing exam, but can also continue their studies to become a Doctor of Pharmacy, which takes an additional 3 years. Other professional diplomas (nurses, midwives, and biomedical technicians) take 3 years [45].

#### Pakistan

In line with its bigger population size, Pakistan has a much larger medical education system than Cambodia, with 114 medical colleges of which 44 are public and 70 are private [46]. The number of graduates from medical colleges has increased from 500 in 1947 at the time of independence to approximately 200 000. This sharp increase is attributed due to the growth of private medical schools from one in 1981 to 32 in 2010 and 55 at present [47]. Concerns about the standards of healthcare professional education, excessive fees charged by private medical colleges, and corruption have resulted in Pakistan’s regulatory body—the Pakistan Medical & Dental Council (PMDC)—being suspended on 20 October 2019, and major reforms are currently underway. We describe the training and licencing system operating since the 1960s (including at the time of the study).

Basic medical training leading to an MBBS degree lasts 5 years. After a 1-year internship (also referred to as a house officer job) in a hospital recognised by the Pakistan Medical & Dental Council (PMDC), and after applying for a medical licence from the PMDC, doctors can work as general practitioners [48]. Registration with the PMDC needs to be renewed every 2 years, but renewal does not require any additional exams, only payment of a fee. Unlike Cambodia, there is no national or provincial exam at the MBBS level. However, the specialist examination for Fellowship of the College of Physicians and Surgeons of Pakistan is a national-level examination. The PMDC documents the minimum content that medical and dental training cover, although regular monitoring of educational institutions has not been sustained.

Pharmacy training takes 5 years, and students are awarded a Doctor of Pharmacy degree upon completion [49]. Like Cambodia, there is no enforcement of compulsory CME, although there are opportunities for CME through the College of Physicians and Surgeons [50].

### Study population

We conducted a total of 85 in-depth interviews with HCPs (doctors, pharmacists, nurses, and medicine sellers), government health agencies, national AMR technical policy advisers, pharmaceutical industry staff, non-governmental organisations working on healthcare, and local representatives of international policy bodies such as the WHO. There was a fairly even balance between countries with 44 interviews in Cambodia and 39 in Pakistan. We used a combination of purposive and snowball sampling to identify interviewees and ensured that all policymakers and technical advisers who were nominated as being influential with respect to policies on access to medicines and regulation of HCP clinical activities were approached for an interview.

### Data collection and analysis

We followed the SRQR reporting guidelines for qualitative studies [51]. Interviews were conducted by two members of the research team, with one Cambodian or Pakistani researcher being involved in each interview. Except in a few cases, where consent for recording was not given, interviews were recorded and detailed interview notes were taken. Interviews were transcribed verbatim and professional translators were used to translate transcripts into English when interviews were conducted in Khmer or Urdu. Participants were de-identified in the transcripts, and we organised and coded each transcript line by line in NVivo (v12). Guided by our conceptualisation of the components of HCP professionalism summarised in Fig. 1, we conducted a thematic analysis employing an interpretive approach in which identified themes are supported by excerpts from the raw data to ensure that data interpretation remains directly linked to the words of the participants [52]. Themes that were identified consistently across both countries were categorised as core themes and are discussed in this paper. After collating our initial findings, we sought feedback from the interviewees and incorporated their input into our analysis.

## Results

Despite the many cultural and sociodemographic differences, there were striking similarities in the dynamics underlying inappropriate use of antimicrobials by HCPs in Pakistan and Cambodia. In line with our hypothesis, we found that there was little reported difference between the practices of ‘unqualified doctors’ (quacks) and qualified doctors with respect to following domestic guidelines on the use of antibiotics, and both qualified pharmacists and unqualified drug sellers routinely dispensed antibiotics without a prescription. Thus, in both countries, the behaviour of many qualified HCPs did not reflect their professional education.

I have seen the biggest quacks are actually doctors themselves. (P_48)

Common examples of inappropriate use of antibiotics by HCPs included the following: for a common cold or diarrhoea, given unnecessarily as prophylaxis during surgery or after normal vaginal delivery, adding multiple antibiotics because of a presumed additive effect on efficacy or because of lack confidence about which antibiotic will work, and incorrect dosing.

Relating our results back to our conceptualisation of HCP professionalism, we first discuss the findings about the two components of competence (technical knowledge on use of antibiotics followed by a collaboration with other HCPs), and then about the two components of care (altruism, professional ethics, and conflict of interest followed by communication).

### Technical knowledge on when and how to use antibiotics imparted during initial HCP education

Ten interviewees in Pakistan and five in Cambodia attributed the widespread inappropriate use of antibiotics partly to weaknesses in the quality of knowledge imparted during HCP education.

First thing that should be done is to send them back to school. Raising awareness. (C_1)

Two critical gaps in the curriculum and training programmes highlighted were inadequate coverage of AMR/microbiology and a scarcity of education on the appropriate use of antibiotics, and lack of attention to preventative measures such as infection control and vaccination. Discussing the latter, some interviewees suggested that HCPs realised that insufficient infection control measures were being taken in health facilities and homes and that this drove them to overuse antibiotics to compensate. We found that the insufficient emphasis on AMR during initial HCP education may be responsible for a perception among HCPs (as reported by them and interviewees who had audited prescribing patterns) that antibiotics can be used for a range of conditions without needing a specific diagnosis and that AMR is not a serious concern because there are other antibiotics available if resistance develops to one:

Definitely, our healthcare professionals are not aware of AMR. They don’t bother, they don’t care about it. To them they have got many options [of antibiotics] (P_35)

Because of the widespread feeling that HCP education is inadequate and that qualified and unqualified HCPs are therefore similar in their technical knowledge, many interviewees felt that qualified HCPs would not always act as good gatekeepers for access to medicines such as antibiotics. There were some differences in the perceived value of HCP education between the two study countries. In Cambodia, four interviewees indicated that qualified HCPs were more aware of the responsibilities around prescribing than non-qualified HCPs. However, in Pakistan, three interviewees said that GPs learn from the incorrect behaviour of specialists and that drug sellers learn from observing the incorrect prescriptions of doctors. Furthermore, one senior clinical microbiologist in Pakistan described a vicious cycle in which hospitals and public health institutions do not want to pay higher salaries to recruit qualified HCPs since they know that the training is inadequate, and this further devalues HCP education institutions (P_20).

### Collaborating with other HCPs

A clear and consistent finding in both countries was the lack of respect for the skills and specific roles of doctors, pharmacists, nurses, midwives, and drug sellers, such that each category of HCPs was taking on the roles of others in order to maximise profits. For instance, there was consensus in both countries that doctors not only diagnose patients but also sell medicines to them; this diminishes the role and income of pharmacists and drug sellers. Pharmacists and drug sellers also encroach upon the doctors’ role by diagnosing patients after listening to their symptoms and then selling medicines that should only be available with a prescription. One interviewee in Pakistan, a government official, expressed concern that pharmacists are not only taking on the roles of doctors in their interactions with patients but that some tell patients that they are medical doctors as their Doctor of Pharmacy degree allows them to use the title of doctor (P_32). A pharmaceutical company employee in Cambodia explained that it is not financially viable for pharmacists to only dispense antibiotics when patients bring a prescription: *If I have my own pharmacy and wait for [a] prescription, then probably I will die* (C_20). We also found, in both countries, that the years of training undertaken by pharmacists often led to them being perceived as ‘overqualified’ to be selling medicines in pharmacies, as this was a role played by drug sellers (with limited professional education); we found that several, but not all, pharmacists used their professional licences to set up pharmacies that they did not work at.

### Altruism, professional ethics, and conflict of interest

Our analysis revealed that a critical element of HCP education—professional ethics and cognisance about conflicts of interest—was inadequate among many HCPs in both countries. It was common for HCPs to run healthcare facilities primarily as businesses, and the influence of the profit-making element of patient consultations on HCPs’ professional judgement was not typically inculcated during training. Therefore, while most HCPs were *not* concerned about conflicts of interest that could drive provider-induced demand for antibiotics, several policymakers believed that this is a major issue that should be addressed by increasing professional ethics training.

Do you think they bother about this AMR? They just bother about their earning. (P_35)

We cannot wait for the law. All the physicians after they finish [professional education], they should have medical ethics. Understand their role. (C_13)

In particular, we documented an entrenched acceptance of gifts and ‘continuing medical education’ from pharmaceutical companies by HCPs, with inadequate questioning of conflicts of interest. The role of pharmaceutical companies in influencing antibiotic use was confirmed by 18 policymakers, HCPs, and pharmaceutical companies in Pakistan and Cambodia. The incentives provided include free medicines (buy 10 get one free), or direct payments/gifts for selling an agreed volume of antibiotics. Although few policymakers and NGO representative referred to the latter as ‘bribes’, they agreed that these unethical practices were common in both countries. Indeed, we found in Pakistan that some companies may have consciously designed incentive packages that counteract questions raised about the ethics of direct incentives provided for selling medicines, by paying for doctors to go on religious pilgrimages to Saudi Arabia.

Another common benefit given to HCPs by pharmaceutical companies, particularly doctors, was access to scientific information about new medicines or a medical condition, either by visiting the doctor’s clinic or by inviting the doctor to an expensive hotel or overseas destination. This strategy often appeared more benign to HCPs than a direct financial incentive for selling an agreed volume of antibiotics. However, it was highlighted that doctors were given trips to attractive destinations—often linked to meeting prescribing targets—under the guise of ‘continuing medical education’. Medical education seminars sponsored by pharmaceutical companies were clearly popular in both countries, partly because they fill a serious gap in the CME opportunities, which were described as extremely limited and under-developed. It was clear from our analysis that HCPs in both countries paid insufficient attention to conflicts of interest that pharmaceutical companies might have when imparting ‘scientific information’.

### Communication

The majority of interviewees in both countries indicated that it is common for patients to expect or demand antibiotics from doctors, pharmacists, and drug sellers. While some HCPs’ inappropriate use of antibiotics stems from their lack of knowledge, as presented earlier, our findings suggest that others know that antibiotics should not be dispensed but have not gained critical communication skills during their training to be able to counsel patients appropriately. Instead, doctors in both countries were reported to feel anxious about being perceived by patients as ‘a D grade doctor’ or about losing future business if they do not give enough medication following a consultation.

Out of 10 consumers, 9 won’t listen to us and they buy what they want. When we advise them, they do not listen to us. Some consumers feel that we want to prescribe drugs for a long duration so that we can make more money (C_29, pharmacist)

A striking finding in both countries was that some HCPs give third-generation antibiotics that patients have not heard of in order to gain patients’ confidence and to appear more knowledgeable. When we presented our initial findings in Cambodia, one clinical and policy advisor on AMR (C_1) strongly emphasised the need for improvements in HCP communication skills to tackle patient demand for antibiotics. Similarly, a well-respected infectious disease specialist in Pakistan stated that lack of HCP communication skills was responsible for the (perceived) strong patient demand for antibiotics:

you know this is an excuse that the most doctors give [for inappropriate antibiotic use] that the patients demand it ...they need your time, they need your explanations and once you secure their confidence they’ll never insist on anything. (P_14).

## Discussion

Based on our findings, we make the potentially contentious assertion that when global guidelines recommend that patients should only seek care at qualified HCPs, it is essential to ensure that HCP qualifications are equipping them to act in the best interest of the patient and society. Our investigation of HCP professionalism, through examining inappropriate use of antibiotics in Pakistan and Cambodia, suggests that this will be a long process. We identified five areas in which strengthening of HCP education could reduce inappropriate use of antibiotics and thereby enhance the social benefits provided by HCPs. These were delivering better teaching about the need for appropriate use of medicines and antibiotics specifically; imparting stronger communication skills to deal with patient demand for antibiotics; inculcating professional ethics; providing skills required for effective collaboration between doctors, pharmacists, and lay HCPs such as drug sellers; and ensuring access to (unbiased) CME. Strengthening of HCP education would also ideally include an emphasis on working across sectors and disciplinary boundaries, for example, to the interconnectedness of human health, animal health, and the environment [53, 54].

The WHO and others have emphasised the social mission of HCP educational institutions [4, 6] and that a crucial part of this culture of neglect is a failure to appreciate the importance of universities as core social institutions that require appropriate regulation [1]. However, it is important to consider whether there are any incentives for HCP education institutions and their students to focus on social accountability, and to assess the extent to which HCPs demonstrate attributes of professionalism, as we do in this study. Concerns have been raised about whether notions about the social mission of healthcare provision may be idealistic and out of touch with the goals of young people who are primarily seeking to earn sufficient income. For example, in Pakistan, a decrease in the proportion of men applying to medical school has been attributed to the relatively low income of doctors [55].

Although strong HCP education that builds upon the components of professionalism we have discussed is essential, we recognise that other dynamics driving inappropriate use of antibiotics are also important to tackle [56]. For example, addressing unethical marketing practices that pharmaceutical companies use to incentivise HCPs is required in addition to a greater emphasis on professional ethics in HCP education [57]. It is also crucial to acknowledge that HCPs often seek additional income or rely on other sources of job motivation because public sector salaries in LMIC are low, and income during clinical training years is typically minimal [58–62]. Finally, low access to affordable diagnostics, and the need for many poor patients earning a daily wage to return to work quickly, will continue to play a role in HCP’s decision-making [17]. Without addressing some of these systemic drivers of inappropriate use of medicines, teaching the values related to professionalism may not translate into behavioural change. Similarly, a conducive regulatory environment is critical, and generating the resources and political will to implement regulations that address poor quality of healthcare is often very challenging [63, 64].

We acknowledge that a limitation of our study is that we were only able to conduct such an in-depth investigation in two countries, and further research would be required to assess the applicability of our findings to other contexts. Our findings about the potential conflicts of interest arising from the relationship between pharmaceutical companies and doctors could be triangulated by non-participant observations at sponsored continuing medical education seminars and during marketing visits by pharmaceutical sales representatives. We appreciate that our views and judgements as researchers may have influenced our interpretations. However, published literature supports our observations, concluding that professional education in many Asian countries has not kept pace with new technologies, societal demands, and health system dynamics related to the growth of informal and private HCPs, because of outdated curricula and poor enforcement of standards [47, 65]. Furthermore, some of the components we identified which require strengthening in HCP education, such as learning communication skills to reassure patients that they do not need an antibiotic, are also relevant to higher-income countries; for example, there was a dramatic reduction in antibiotic prescribing when GPs in the Netherlands received training in enhanced communication skills [66], and a systematic review of physician-targeted interventions to improve antibiotic use for respiratory tract infections identified communication skills training as the most promising [67].

## Conclusion

It is often assumed that increasing access to qualified HCPs will be beneficial to patients and to society because qualified HCPs will demonstrate more competence and care than unqualified HCPs when delivering clinical services. Our study provides evidence from two diverse countries indicating that HCP professional education may not be equipping HCPs with essential skills and values to ensure that they act in the patient’s best interest when providing antibiotics. Although HCP educational institutions are not presently held to account for the ways in which their graduates serve their societies, we question whether such accountability should be required, particularly in light of the increase in private medical institutions in many LMIC. Finally, we conclude that the specific improvements to HCP education that we have identified are particularly pertinent for LMIC contexts, since self-regulation of HCPs plays a critical role when government ability to enforce rules is lacking [68, 69].

## Data Availability

The datasets generated and/or analysed during the current study are not publicly available but are available from the corresponding author on reasonable request.

## Abbreviations

AMR: Antimicrobial resistance
CMC: Cambodia Medical Council
CME: Continuing Medical Education
HCP: Healthcare provider
LMIC: Low- and middle-income country
PMDC: Pakistan Medical and Dental Council
